# Photoluminescence of 2D and 3D quantum dots synthesized by laser-ultrasonic treatment on van der Waals materials

**DOI:** 10.1039/d5na00885a

**Published:** 2025-11-04

**Authors:** Alexei V. Prokhorov, Anton S. Chernikov, Gleb I. Tselikov, Alexander V. Shesterikov, Mikhail Yu. Gubin, Ivan S. Kazantsev, Alexander V. Syuy, Ilya A. Zavidovskiy, Elena S. Zhukova, Anton A. Popov, Kirill S. Khorkov, Dmitry A. Kochuev, Aleksey V. Arsenin, Valentyn S. Volkov

**Affiliations:** a Department of Physics and Applied Mathematics, Vladimir State University named after Alexander and Nikolay Stoletovs 600000 Vladimir Russia; b Emerging Technologies Research Center, XPANCEO 00000 Dubai United Arab Emirates alprokhorov33@gmail.com; c Moscow Center for Advanced Studies 123592 Moscow Russia; d Department of General Physics, Perm National Research Polytechnic University Perm 614990 Russia; e National Research Nuclear University MEPhI 115409 Moscow Russia

## Abstract

A two-stage method for synthesis of van der Waals quantum dots in a liquid by laser ablation of the initial material followed by ultrasonic treatment of the samples is proposed and implemented. The use of various initial samples in the form of pressed powder targets or transition metal dichalcogenide crystals allows the fabrication of both three-dimensional and flat few-layer quantum dots that are capable of exhibiting a photoluminescent response in a wide spectral range. The possibility of straightforward control of photoluminescence spectra in ensembles of MoS_2_, WS_2_, MoSe_2_, and WSe_2_ quantum dots with wide-size dispersions by tuning the pump laser wavelength is shown.

## Abstract 2

Ensembles of van der Waals quantum dots with a wide-size dispersion, fabricated by two-stage laser and ultrasonic treatment, exhibit the pump-dependent photoluminescence in the visible spectral range.

## Graphical abstract

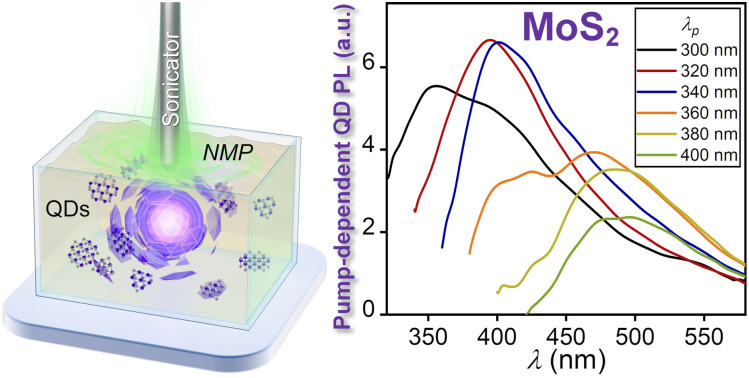

## 1 Introduction

At one time, the synthesis of quantum dots (QDs) in the form of bulk nanocrystals opened new horizons for experimental quantum physics and photonics.^1,2^ The introduction of new highly anisotropic materials into laboratory practice using the layered van der Waals (vdW) materials has raised non-trivial questions about reliable methods for obtaining quantum dots based on them and fundamental properties of their photoluminescence.^3^ Fundamentally, the nature of quantum confinement in vdW QDs is inherently anisotropic and can manifest in two distinct morphological archetypes.^4^ The first consists of planar, few-layer QDs (2D QDs), where confinement is strong in the out-of-plane direction (defined by the layer thickness) and tunable in the in-plane direction (defined by lateral dimensions). These structures are expected to exhibit PL across various sizes, with the emission energy governed by lateral confinement.^5,6^ The second archetype comprises quasi-spherical, polycrystalline nanocrystals (3D QDs). In these structures, the bulk vdW material's indirect bandgap persists unless the overall size is reduced to a few nanometers, commensurate with only a few atomic layers, where the band structure begins to resemble that of a monolayer.^7,8^ The ability to selectively synthesize these distinct archetypes is critical, as the dimensionality of confinement dictates their fundamental optoelectronic behavior and suitability for specific applications, ranging from active flat optics (2D)^9,10^ to theranostics (3D).^11–13^

Precise synthesis control over these morphologies is crucial for harnessing their distinct optical properties.^4^ Current synthesis methods, including modified liquid-phase exfoliation,^14^ and laser ablation of TMDC powders or crystals,^15–18^ often yield ensembles with ambiguous morphologies or require complex chemical procedures. Femtosecond laser ablation in liquids (LAL) is a versatile technique for nanoparticle synthesis; however, high-energy exposure can induce non-equilibrium processes, such as coulomb explosion, leading to the formation of QDs.^8,11,19^ Secondary treatments, such as ultrasonication (US), have been employed to exfoliate these nanoparticles or fragment bulk materials into planar QDs.^20,21^ Yet, a deterministic approach to selectively synthesize either 2D or 3D vdW QDs remains elusive.

**Fig. 1:**
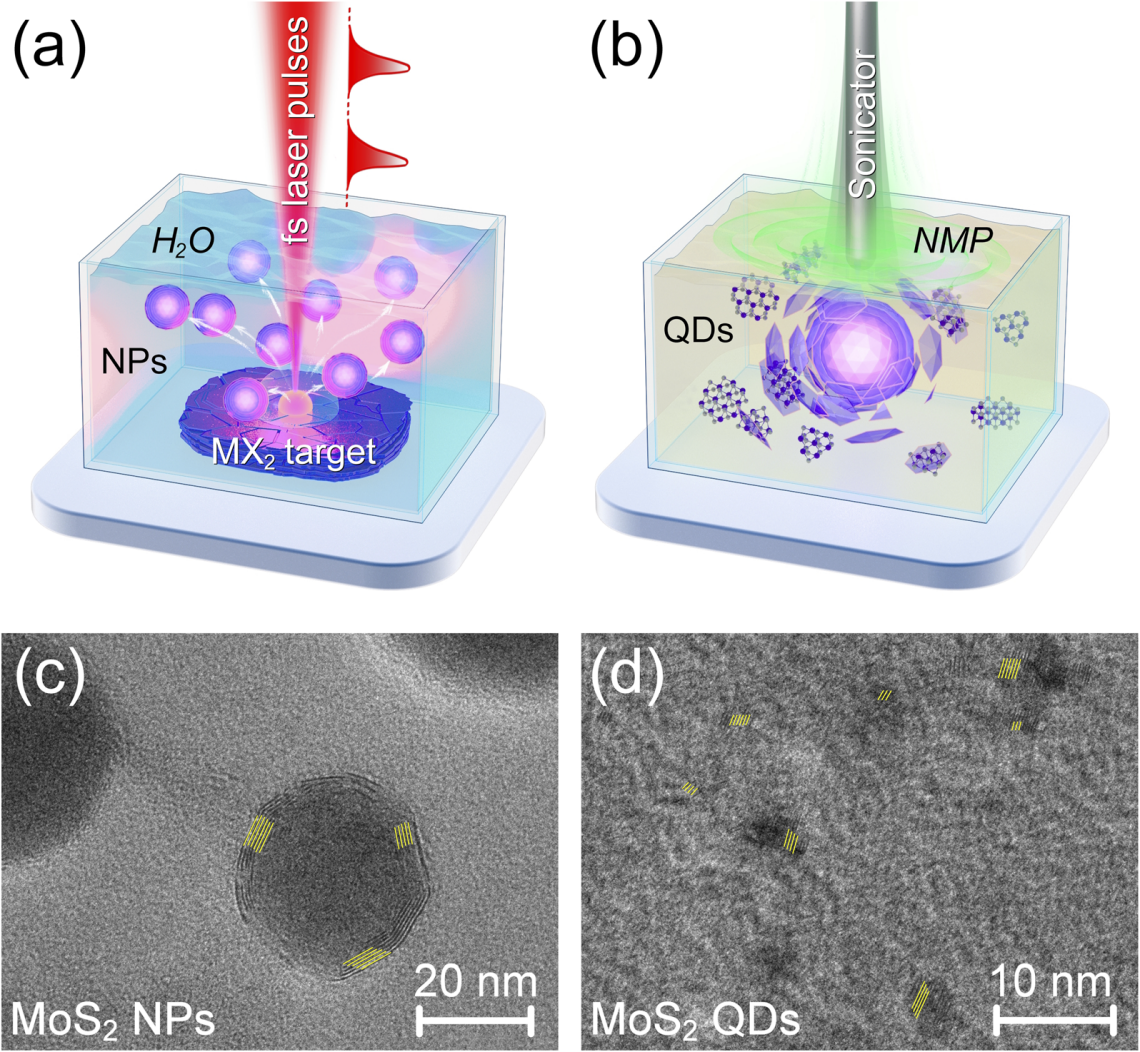
Concept of two-stage synthesis of nanostructures using (a) femtosecond laser ablation of the target placed in water followed by transfer of NPs to NMP and centrifugation for subsequent (b) ultrasonication. TEM images of (c) core–shell NPs synthesized by laser ablation of the MoS_2_ target in water and (d) MoS_2_ QDs obtained after transfer of NPs to NMP and sonication. The average distances between lattice fringes in panels (c) and (d) are 0.61 nm and 0.28 nm, respectively.

In this work, we applied a two-stage method for the synthesis of vdW QDs with various shapes using femtosecond laser ablation followed by ultrasonic exposure on various samples of transition metal dichalcogenides, “fs+US” treatment, as shown in Fig. 1a and b. We put forward and validate the hypothesis that the physical form of the initial bulk TMDC target, namely, low-density pressed powder *versus* a high-density monolithic crystal, can serve as a macroscopic adjustor to determine the final, nanoscale morphology of the obtained QDs, yielding either planar 2D flakes or quasi-spherical 3D nanocrystals. To characterize the obtained QDs and reveal their features, we employed the methods of Raman and infrared spectroscopy, transmission electron microscopy, and the numerical analysis of experimentally measured photoluminescence spectra. The developed approaches and methods can be used for the synthesis of photoluminescent vdW QDs with given spectral features.

## 2 Results and discussion

### 2.1 Synthesis and characterization of quantum dots

At the initial stage, we studied the influence of high-energy exposure on the solutions of vdW materials, as shown in Fig. 1a. A femtosecond Yb:KGW laser system TETA-10 (Avesta Project Ltd, Russia) was used as a radiation source, which provides the emission at a wavelength of 1030 nm with a pulse duration of 280 fs and pulse repetition rate of 10 kHz. Laser beam scanning was carried out using a galvanometer scan head RLA-1504 (RAYLASE GmbH, Germany); beam focusing was performed using an F-Theta lens with a working distance of 200 mm, and the beam diameter in the caustic region was about 70 µm. The samples of indirect band gap transition metal dichalcogenides were used as the starting material for treatment. For all materials, the laser ablation synthesis was carried out in deionized water; the samples were placed at the bottom of a cuvette filled with 10 ml of liquid, and the height of the liquid above the target surface was 4 mm. The pulse energy for ablation of MoS_2_ and WS_2_ materials was 50 µJ, and the fluence value *F*_ab_ was 2.6 J cm^−2^. For ablation of MoSe_2_ and WSe_2_ materials, the pulse energy was 100 µJ with a fluence of 5.2 J cm^−2^. For MoS_2_ and WS_2_ samples, the treatment area was a square with a side of 4 mm, the density of scanning tracks was 20 lines per mm, the scanning speed was 100 mm s^−1^, and the process duration is 3 minutes. For MoSe_2_ and WSe_2_ samples, the treatment area was a square with a side of 2 mm, the density of scanning tracks was 20 lines per mm, the scanning speed was 20 mm s^−1^, and the process duration is 10 minutes. Note that the ablation thresholds *F*_th_ for the used materials are 0.05, 0.3, 0.09, and 0.01 J cm^−2^ corresponding to MoS_2_, WS_2_, MoSe_2_, and WSe_2_, respectively. Therefore, the coefficients *k* = *F*_ab_/*F*_th_, showing the relation between fluence and threshold values, are 52, 8.67, 57.78, and 520 for MoS_2_, WS_2_, MoSe_2_, and WSe_2_, respectively.

The use of various samples in the form of both pressed powder and crystals for laser ablation facilitated the synthesis of nanoparticles with different morphologies in the first step, as shown in Fig. S1.1. In particular, the use of pressed powder targets for ablation resulted in the formation of relatively friable MoS_2_ and WS_2_ nanoparticles with concentric arrangement of layers, as shown in Fig. 1c and S1.1b for such MoS_2_ NPs.

These types of NPs can be obtained even by ablation from lower-density crystals of MoS_2_ and WS_2_ materials,^22^ and the use of pressed powder additionally reduces the density of the target material to form a thicker concentric-layered shell, as shown in Fig. 1c. In contrast, the higher-density crystals of MoS_2_, MoSe_2_ and WSe_2_ materials provide the formation of only heavy polycrystalline NPs with various sizes without even a small shell. A comparison of NPs with similar sizes, fabricated from different materials and target types, is provided in Figs. S1.1a, b, and d. Note that in the case of laser exposure on the MoSe_2_ crystal with the ratio *k* similar to the one of the MoS_2_ material, the formation of a wide variety of NPs is observed, including crystallites shown in Fig. S1.1 and even spherenes, *i.e.*, particles consisting entirely of concentric-layered shells.

At the second stage, the ultrasonic exposure on the obtained colloidal solutions was performed, as shown in Fig. 1b. At this stage, *N*-methyl-2-pyrrolidone (NMP) was chosen as a high-fluidity compound with a small size of molecules. The intermediate stage was the centrifugation of colloidal solutions obtained by laser ablation synthesis in order to sediment NPs and their subsequent transfer to NMP. The stability of the nanoparticles during this solvent exchange relies on the inherent properties acquired during laser ablation; NPs synthesized *via* LAL typically possess a significant surface charge, providing electrostatic stabilization.^23^ Centrifugation was carried out in centrifuge tubes (1 ml of solution in each tube) using a microcentrifuge D3024 (DLAB Scientific Co., Ltd) at the relative centrifugal force (RCF) 10 000×*g*, and the process duration is 15 minutes. After the centrifugation, the supernatant was collected in such a way that the minimum possible amount of a liquid remains in the test tube. Next, 1 ml of NMP was immediately added to each test tube. To prevent agglomeration and ensure efficient redispersion of the “soft” pellet, the test tubes were promptly placed in a low-power ultrasonic bath for 2 minutes. Thus, the deionized water was replaced with NMP. The obtained solutions of NPs in NMP were transferred to 10 ml glass test tubes. Ultrasonic exposure on colloidal solutions was carried out by means of a probe sonicator USTA-0,1/28-O (U-Sonic, Biysk, Russia) with an intensity of US exposure of 240 W cm^−2^. The probe was immersed in the test tube to a depth of 50 mm. To avoid overheating during US treatment, the glass test tube was placed in a bath filled with a cold water and ice. The duration of US treatment was 30 minutes.

The results of ultrasonic exposure on the solutions obtained after laser ablation significantly depend on the solutions of ablated NPs. Ultrasonic treatment of the solutions obtained from the crystals did not result in the modification of NPs, but contributed to their characteristic agglomeration, as shown in Fig. S1.2a. At the same time, such agglomerates could contain both fairly large NPs and smaller 3D QDs. Moreover, the similar US treatment of solutions obtained from MoS_2_ pressed powder targets led to the appearance of a large number of small particles, *i.e.*, quantum dots with a planar alignment of layers similar to that in Fig. 1d and S1.2b. We assume that the appearance of such flat 2D QDs can be due to the destructive effect of ultrasound on the shells of large concentric-layered NPs in NMP, as shown in Fig. 1b and d. This is also verified by the measured characteristic distances between lattice fringes of 0.61 nm in the shell of MoS_2_ NPs immediately after femtosecond laser treatment as shown in Fig. 1c and 0.28 nm in QDs after US treatment as shown in Fig. 1d. In the former case, this distance corresponds to the interlayer distance in MoS_2_, and in the latter case, it corresponds to the interplanar spacing for this material. In other words, when the concentric-layered shells break down, the small planar fragments, *i.e.*, quantum dots, are formed. The “fs+US” treatment of the MoS_2_ target yields solutions containing both flat 2D QDs and residual large NPs, as evidenced in Fig. S1.3b. Thus, the final results of the two-stage treatment of various samples are the solutions containing both the large NPs in all fractions and smaller QDs of various types. We are talking about a small number of 3D (spherical) QDs in solutions obtained from the initial crystals, as well as the presence of flat 2D QDs in solutions prepared from pressed powder targets.

**Fig. 2:**
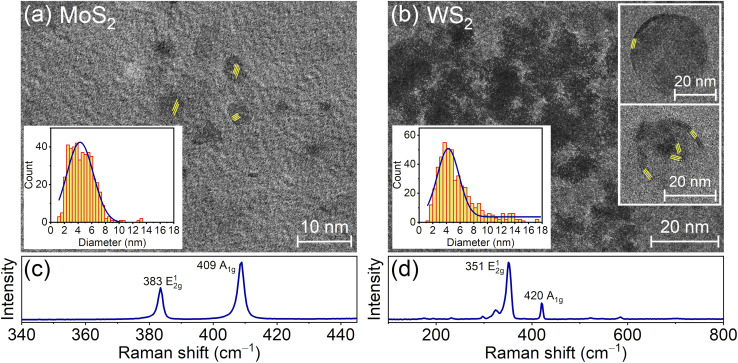
(a and b) TEM images of NPs obtained by the complex method of laser ablation in water and subsequent ultrasonic (fs+US) treatment in NMP from pressed powder targets made of (a) MoS_2_ and (b) WS_2_ materials. (c and d) Raman spectra of drop-cast solutions presented in panels (a) and (b). The left insets of panels (a) and (b) show the histograms of size distributions for the obtained NPs and right insets of panel (b) show the images of shells pulled from nanoparticles (bottom inset), as well as the forming QD (top inset). The average distances between lattice fringes in panel (a) and in the insets of panel (b) are 0.28 nm and 0.62 nm, respectively.

**Fig. 3:**
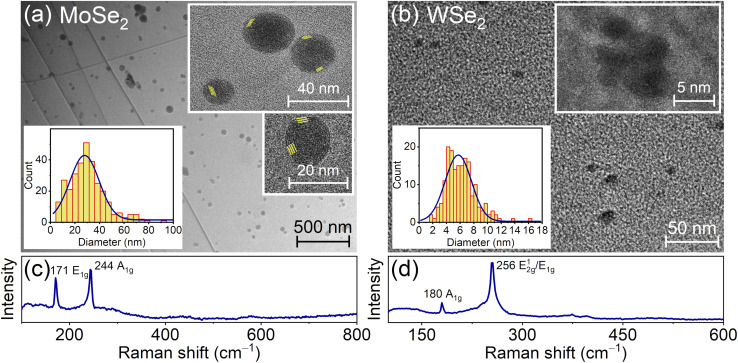
(a and b) TEM images of NPs obtained by the complex method of laser ablation in water and subsequent ultrasonic (fs+US) treatment in NMP from (a) MoSe_2_ and (b) WSe_2_ crystals. (c and d) Raman spectra of solutions presented in panels (a) and (b). In panels (a) and (b), the left insets show the histograms of size distributions for the obtained NPs and right insets show the images of nanoparticles and 3D QDs. The average distance between lattice fringes in the insets in panel (a) is 0.68 nm.

The detailed analysis of transmission electron microscopy (TEM) images of NPs in solutions obtained by two-stage treatment based on pressed powder MoS_2_ and WS_2_ targets, as well as based on MoSe_2_ and WSe_2_ crystals, shown in Fig. 2 and 3, revealed the presence of NP distributions with wide-size dispersion for all target materials. Based on the analysis of NP size distributions in the left insets of Fig. 2, the average sizes of MoS_2_ and WS_2_ NPs are 4.4 nm and 4.7 nm, respectively, whereas for MoSe_2_ and WSe_2_ NPs in Fig. 3 these parameters are 29.7 nm and 6.1 nm, respectively. Raman spectroscopy was used to analyze the chemical composition of the obtained NPs and QDs, as shown in Fig. 2c, d and 3c and d. Based on Raman analysis, it can be assumed that the MoS_2_ and WS_2_ particles in the solutions in Fig. 2 are in the crystalline phase. Besides, the characteristic distance between peaks indicates the predominant presence of a bulk material, which corresponds to the response of large bulk NPs dominating in the solution, as shown in histograms in the insets of Fig. 2a and b.

At the same time, TEM images of single QDs shown in Fig. 2a clearly indicate the presence of small MoS_2_ quantum dots with planar nature. Moreover, the right insets of Fig. 2b show the large fragments of shells pulled from core-shell WS_2_ nanoparticles during US exposure on them. Furthermore, it is possible to distinguish the pieces detached from the shells, which can become QDs.

In contrast, after fs+US treatment, nanoparticles made of selenide crystals remain spherical and either retain a concentric geometry, as for the MoSe_2_ material in Fig. 3a, or become polycrystalline nanoparticles of various sizes, as for the WSe_2_ material in Fig. 3b. Note that, according to the histograms in Fig. 3a and b, the average sizes of the obtained MoSe_2_ NPs are significantly larger than those of WSe_2_ NPs with almost the same numbers of 3D QDs for these two materials. The point is that the two processes can occur during laser exposure on dense crystals. At low pump fluence, thermodynamic equilibrium melting with evaporation and formation of large NPs is realized. At high pump fluence, a nonequilibrium phase (Coulomb) explosion can occur, which leads to the formation of small QDs.^19,24^ Apparently, under exposure on a moving crystal (the use of a magnetic stirrer), its illuminance changes over time even at a fixed pump fluence. Therefore, the formation of NPs and QDs occurs simultaneously. At the same time, the ratios between numbers and sizes of NPs and QDs can be determined by the exceptional properties of the used crystals. In particular, for the same sizes and numbers of QDs, the yields and sizes of NPs can be different, which can be clearly seen for the comparison of MoSe_2_ and WSe_2_ materials in Fig. 3. Besides, in the case of the MoSe_2_ material, the large thickness of the shell in combination with the used power of US exposure does not allow the breakdown of NPs up to the QD state in contrast to the case shown in Fig. 2. In the case of the WSe_2_ material, the NPs possess a polycrystalline structure immediately after femtosecond laser treatment and retain it after US exposure. To verify the chemical composition of the obtained NPs and QDs, we preformed the energy dispersive X-ray analysis of the solutions, which confirmed the agreement between chemical compositions of particles and initial samples for ablation, as shown in Fig. S1.3.

In general, fs+US treatment of vdW materials in a liquid leads to the formation of both vdW NPs and 2D and 3D vdW QDs in the solution. However, even without any separation, the photoluminescence properties of such solutions are determined solely by the concentration and morphological features of quantum-sized particles, including multilayer flakes of considerable area.

### 2.2 Photoluminescence control in QD ensembles with a wide-size dispersion

**Fig. 4:**
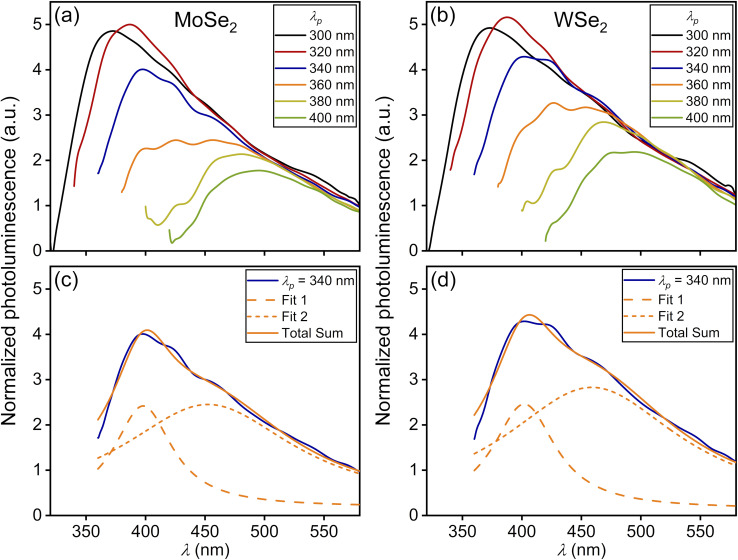
Photoluminescence spectra for an ensemble of 3D QDs with a wide-size dispersion excited by various pump wavelengths *λ*_p_ = 300, 320, 340, 360, 380, 400 nm and exhibiting the corresponding photoluminescence maxima *λ*_PL_ (with QD sizes *D*_QD_ = 2*R* calculated according to the formula (1)) for solutions obtained using the two-stage method fs + US on the basis of the (a) MoSe_2_ material with *λ*_PL_ = 373, 387, 398, 457, 481, 495 nm (*D*_QD_ = 1.73, 1.78, 1.82, 2.03, 2.12, 2.18 nm) and (b) WSe_2_ material with *λ*_PL_ = 373, 388, 402, 427, 469, 495 nm (*D*_QD_ = 1.84, 1.90, 1.95, 2.05, 2.23, 2.35 nm). (c) and (d) Deconvolution of photoluminescence spectra for MoSe_2_ and WSe_2_ QDs, respectively, under excitation by pump wavelength *λ*_p_ = 340 nm.

**Fig. 5:**
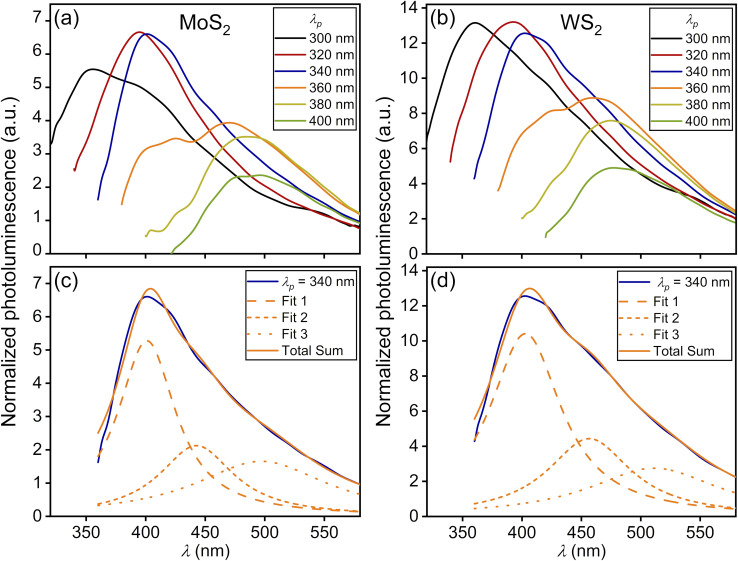
Photoluminescence spectra for an ensemble of 2D QDs with a wide-size dispersion excited by various pump wavelengths *λ*_p_ = 300, 320, 340, 360, 380, 400 nm and exhibiting the corresponding photoluminescence maxima *λ*_PL_ (with QD sizes *D*_QD_ = 2*ρ*_0_ calculated according to the formula (3)) for solutions obtained using the two-stage method fs + US on the basis of the (a) MoS_2_ material with *λ*_PL_ = 356, 395, 401, 471, 488, 498 nm (*D*_QD_ = 1.54, 1.72, 1.75, 2.17, 2.30, 2.38 nm) and (b) WS_2_ material with *λ*_PL_ = 361, 393, 403, 459, 475, 477 nm (*D*_QD_ = 1.84, 2.06, 2.14, 2.69, 2.90, 2.93 nm). (c) and (d) Deconvolution of photoluminescence spectra for MoS_2_ and WS_2_ QDs, respectively, under excitation by pump wavelength *λ*_p_ = 340 nm.

The study of the photoluminescence properties of the prepared solutions was carried out using a Varioskan LUX Multimode Microplate Reader (Thermo Fischer Scientific, USA). A double monochromator scheme was used for excitation control and emission analysis. The excitation wavelength was varied in the range from 300 nm to 400 nm while the detection range of the PL signal was from 325 nm to 600 nm. The QD solutions were transferred to 96-well plastic plates with a UV-transparent well bottom prior to the PL spectral measurements. First, we consider the general properties of QD photoluminescence, which are independent of their morphology and the type of vdW material used for their fabrication. It is known that the spectral position of the PL peak of the QD ensemble with a narrow-size dispersion is almost independent of the pump wavelength *λ*_p_, while its intensity first increases and then decreases with decreasing *λ*_p_.^14,17^ However, for QD ensembles with a wide-size dispersion, as shown in Fig. 2 and 3, the PL peak position shows significant variation with pump wavelength, as shown in Fig. 4 and 5. The pump-dependent PL behavior in QDs originates from size-tunable interband transitions governed by quantum confinement effects.^25^ Regardless of the QD type, the energy of the interband transition increases upon decreasing the QD size.^26,27^ Therefore, if the pump wavelength *λ*_p_ is greater than the wavelength *λ*_IB_ of the main 1S(e) → 1S(h) transition for all QD subsets in the ensemble, then such an ensemble is not excited and, consequently, does not emit the light, as shown in Section S2.5. As soon as the pump wavelength becomes less than *λ*_IB_ of the largest emitters in the ensemble, it begins to emit photoluminescence. A further decrease in *λ*_p_ results in engaging of more and more new subsets of QDs with smaller sizes. Thus, the shorter PL wavelengths begin to contribute to the PL process, which leads to a blue shift of the maximum of the PL spectrum, as shown in Fig. 4a, b and 5a and b.^5,14,20^ In this case, each pump wavelength can be associated with the most probable QD size, which mainly contributes to the photoluminescence, as shown in Table S3.1. However, the resulting PL spectrum is formed by not only such QDs with the maximum contribution to PL, but also all other QD fractions with larger sizes that determine the shape of the long-wavelength spectral range. As a result, all PL spectra for QD ensembles with a wide-size dispersion in Fig. 4 and 5 have very asymmetric shapes with steep short-wavelength and gently sloping long-wavelength sides. At the same time, there is an actual limitation on the minimally available QD size in each ensemble, which together with a decrease in the quantum yield for the short-wavelength pump^14^ leads to a decrease in PL intensity under the *λ*_p_ < 360 nm condition for all spectra presented in Fig. 4 and 5.

Now we focus on the characteristic features that are inherent in QDs made of indirect band gap transition metal dichalcogenides being considered in this work. As can be seen in Fig. 3, the MoSe_2_ and WSe_2_ particles are 3D particles possessing a spherical shape.

The energy of the main interband transition in 3D QDs with a conventional spherical shape can be estimated using the formula (see Section S2)1
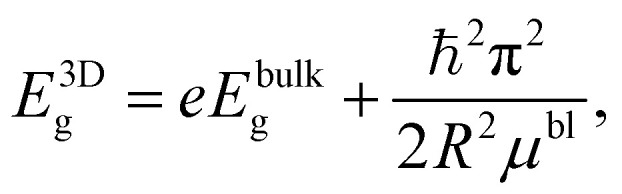
where 
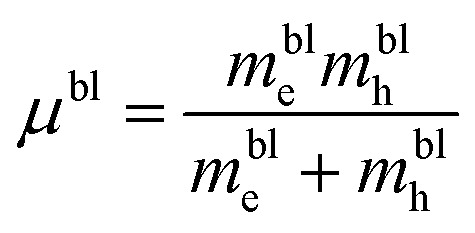
 is the reduced mass of an electron and hole, *m*^bl^_e(h)_ is the effective mass of an electron (hole) for an isotropic (bulk) material, *E*^bulk^_g_ is the bulk band gap for a QD material, *R* is the sphere radius, and *e* is the absolute value of electron charge, as shown in Section S2.3. However, the expression (1) is formally insensitive to the features of the interband transition in the wave-vector space. Both concentric MoSe_2_ and polycrystalline WSe_2_ particles exhibit light emission only below a critical diameter corresponding to 4–5 atomic layers, *i.e.*, when the transition actually becomes direct band gap in its nature.^6^ Thus, only the smallest particles of the entire ensembles of MoSe_2_ and WSe_2_ NPs with wide-size dispersions, which correspond to the left side of histograms for the NP size distributions in the insets of Fig. 3, are able to emit photoluminescence. In this regard, the total PL intensity of the ensemble of MoSe_2_ and WSe_2_ QDs is not so high, and the position of the long-wavelength side of the spectra in Fig. 4 is insensitive to the changes in pump wavelength.

Since the evaluated average size of 3D QDs emitting photoluminescence under excitation at pump wavelength *λ*_p_ = 400 nm takes the value 2.18 nm for MoSe_2_ and 2.35 nm for WSe_2_, the larger QDs, being indirect-band-gap QDs, are almost not engaged in the photoluminescence. For the mathematical description of QD PL, we use the deconvolution method^28,29^ with a set of Lorentz distribution functions for the intensity in the form:2
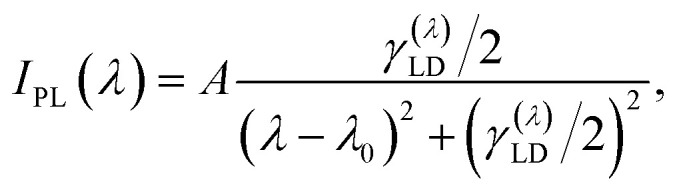
where *λ*_0_ is the central wavelength and *γ*^(*λ*)^_LD_ is the full width at half maximum (FWHM) in the wavelength scale, and *A* is the fitting parameter. Note that the calculation of the spontaneous relaxation rate for the considered QDs is presented in Section S2.8. However, we do not use these findings in our simulations because they do not take into account ensemble effects. At the same time, a clear tendency to increase the relaxation rate for single 2D QDs can be exploited for their coupling to planar photonic structures. For the case of PL from the QD ensembles with a wide-size dispersion pumped at a wavelength of 340 nm, the PL spectra for MoSe_2_ and WSe_2_ QDs are well approximated by using two functions (2) with the parameters presented in Table S3.2. Moreover, the Lorentz distribution function at a wavelength of 398 nm (402 nm) for MoSe_2_ (WSe_2_) QDs with an average size of 1.82 nm (1.95 nm) well approximates the short-wavelength side of the PL spectrum, as shown in Fig. 4c and d. At the same time, the second function peaking at a wavelength of 452.6 nm (458.8 nm) for MoSe_2_ (WSe_2_) QDs corresponds to the PL from larger QDs with an average size of 2.02 nm (2.19 nm), which confirms the PL behavior for the ensemble with a wide-size dispersion.

Finally, the obtained sulfide-based 2D MoS_2_ and WS_2_ QDs exhibit a slightly different size dependence of the 1S(e) → 1S(h) transition compared to the 3D case (see Section S2):3
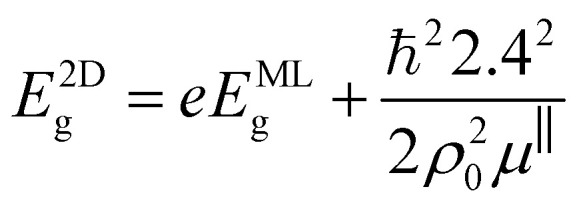
where 
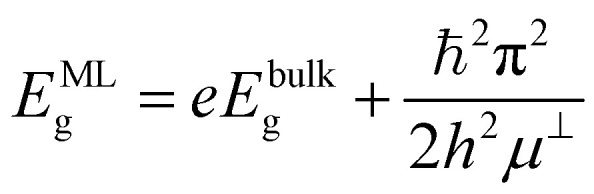
 is the band gap of the infinite monolayer, 
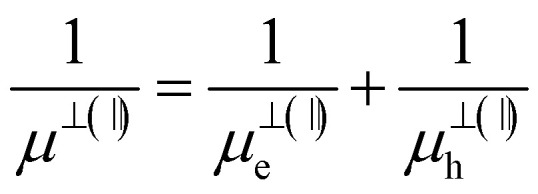
 is the reduced effective mass, *m*^⊥(‖)^_e_ (*m*^⊥(‖)^_h_) is the effective mass of an electron (hole) across (⊥) and along (‖) layers of the material, and *ρ*_0_ and *h* are the cylinder radius and height, respectively, if we consider a QD with cylindrical shape, as shown in Section S2.4. These anisotropic quantum emitters exhibit dual quantum confinement: one arising from their monolayer/few-layer thickness, and another from their in-plane QD dimensions. These few-layer QDs exhibit photoluminescence across all size scales, from nanoscale regions to macroscopic flakes, with emission corresponding to their band gap transition.^6^ Note that in further calculations we consider *E*^ML^_g_ as the energy of the interband transition of the monolayer, as shown in Table S2.4.

Fig. 5 shows that the PL curves of MoS_2_ and WS_2_ QDs for various pump wavelengths do not form bundles of closely spaced curves at the long-wavelength side in addition to the increase in maximum PL intensity, which is especially noticeable for 2D WS_2_ QDs. Actually, this is due to the efficient pump-dependent photoluminescence of large-area 2D NPs that mainly contribute to this part of spectra. Remarkably, the deconvolution method for 2D QDs yields results in good agreement between theory and experiment when fitting by using an already larger number of functions of type (2) with increasing central wavelengths, as shown in Fig. 5c and d, and Table S3.3. This verifies the fact of photoluminescence of large 2D QDs in the long-wavelength region.

It should be noted that US exposure in NMP can lead to both the formation of surface and edge defects in QDs,^30^ as well as QD functionalization with carbon-based groups induced by laser-assisted solvent-QD interaction.^16,20^ These effects can contribute to pump-dependent behavior of the PL spectra of QDs. However, the complementary infrared spectroscopy studies of the solutions obtained after US treatment indicate the minimal or negligible functionalization of luminescent QDs, as shown in Section S5.

On a final note, the synthesis of vdW QDs with tunable photoluminescence requires further study using the entire variety of vdW materials. Further research will focus on identifying clear criteria for sample preparation and energy impacts on the samples in order to obtain chromophores with a given morphology and size. In general, the differences between the PL spectra of 2D and 3D QD ensembles in the far-field region are not so significant, especially for the ensembles with a wide-size dispersion as in this work. However, the fundamental distinction lies not only in their spectral behavior (*e.g.*, the indirect gap threshold in 3D QDs) but also in the anisotropy of their emission at the single-emitter level. The planar morphology of 2D QDs results in highly anisotropic transition dipole moments, predominantly oriented in-plane. This leads to distinct, directional radiation patterns compared to the relatively isotropic emission of 3D QDs.^31–34^ At the same time, the photoluminescent QDs possessing various morphologies may have considerably different applications. For example, the ability of 3D QDs to easily penetrate the bloodstream could be useful for theranostic applications.^11,12^ However, the ability of 2D QDs to be simply integrated with planar interfaces may find application in active flat optics, including active imaging lenses,^9^ microlasers and luminescent metamaterials,^35–40^ as well as integrated plasmonic devices^41–43^ and single photon sources.^34,44^ In the latter case, the use of single 2D QDs requires a detailed study of the near-field response and radiation patterns of such strongly anisotropic quantum emitters, including adaptation of methods of near-field diagnostics.^45^

## 3 Conclusion

In this work, we developed and implemented the two-stage method of fs+US treatment for the fabrication of QD ensembles with a wide-size dispersion from indirect band gap TMDCs. Using transmission electron microscopy and Raman spectroscopy methods for characterization of materials, the chemical composition and topology of nanoparticles and quantum dots obtained from pressed MoS_2_ and WS_2_ powder targets, as well as MoS_2_, MoSe_2_ and WSe_2_ crystals were investigated. It was found that quantum dots obtained from pressed powder targets have a crystalline structure and planar morphology (2D QDs). At the same time, QDs obtained from crystals can have both a polycrystalline structure and non-planar topology (3D QDs).

As a result, all the obtained quantum emitters demonstrate prominent photoluminescence, and control of the pump wavelength in an ensemble of chromophores with a wide-size dispersion allows us to tune the maximum photoluminescence to the desired spectral range. At the same time, 2D QDs obtained from pressed powder targets exhibit sensitivity to the pump wavelength over a wide spectral range, whereas long-wave photoluminescence of 3D QDs is insensitive to the pump. The observed behavior arises from a critical size threshold for 3D TMDC emitters, beyond which the transition to an indirect band gap quenches photoluminescence. A promising evolution of this methodology is the integration of ultrasonic and laser exposures in a single reactor, enabling simultaneous treatment. This “*in situ*” sono-laser approach could potentially enhance synthesis efficiency and yield through the synergistic interaction of laser ablation and acoustic cavitation. We believe that the further combination of ultrasonic and laser exposures in one test tube, with the possibility of their parallel use, as well as the use of the entire class of van der Waals materials, can become the cornerstone of the technique for obtaining high-purity two-dimensional emitters with tunable photoluminescence spectra. In general, such bright anisotropic quantum emitters have broad prospects for practical use in lasing, imaging, sensing, and laser theranostics.

## Author contributions

Alexei V. Prokhorov: conceptualization, project administration, formal analysis, writing-original draft, writing-review & editing, methodology. Anton S. Chernikov: investigation, formal analysis, writing-original draft. Gleb I. Tselikov: investigation, project administration, writing-original draft, writing-review & editing. Alexander V. Shesterikov: data curation, software. Mikhail Yu. Gubin: conceptualization, formal analysis, visualization, writing-original draft, writing-review & editing. Ivan S. Kazantsev: investigation. Alexander V. Syuy: investigation. Ilya A. Zavidovskiy: investigation. Elena S. Zhukova: investigation. Anton A. Popov: investigation. Kirill S. Khorkov: investigation, funding acquisition, validation. Dmitry A. Kochuev: investigation. Aleksey V. Arsenin: conceptualization, project administration, funding acquisition, validation, resources. Valentyn S. Volkov: conceptualization, project administration, funding acquisition, validation, writing-original draft, writing-review & editing, supervision.

## Conflicts of interest

There are no conflicts of interest to declare.

## Supplementary Material

NA-OLF-D5NA00885A-s001

## Data Availability

The data supporting this article have been included as part of the supplementary information (SI). Supplementary information: Additional experimental data and description of the used methods and equipment. See DOI: https://doi.org/10.1039/d5na00885a.
