# Supplementary material for: Photoluminescence of 2D and 3D quantum dots synthesized by laser-ultrasonic treatment on van der Waals materials
Source: Nanoscale Adv. 2025 Nov 4;8(1):135–44. doi: 10.1039/d5na00885a (PMC12631796; doi:10.1039/d5na00885a)
Supplement: NA-008-D5NA00885A-s001 [file NA-008-D5NA00885A-s001.pdf]

Supplementary Information (SI) for Nanoscale Advances.
This journal is © The Royal Society of Chemistry 2025

Supplementary Information for Photoluminescence of 2D and 3D quantum dots synthesized by laser-ultrasonic treatment on van der Waals materials

Alexei V. Prokhorov[*1,2,3], Anton S. Chernikov[1], Gleb I. Tselikov[2], Alexander V. Shesterikov[1,3], Mikhail Yu. Gubin[1,3], Ivan S. Kazantsev[2], Alexander V. Syuy[2,3,4], Ilya A. Zavidovskiy[3], Elena S. Zhukova[3], Anton A. Popov[5,1], Kirill S. Khorkov[1], Dmitry A. Kochuev[1], Aleksey V. Arsenin[2], and Valentyn S. Volkov[2]

[1]*Department of Physics and Applied Mathematics, Vladimir State University named after Alexander and Nikolay Stoletovs, Vladimir 600000, Russia*
[2]*Emerging Technologies Research Center, XPANCEO, Emmay Tower, Internet City, Dubai 00000, United Arab Emirates*
[3]*Moscow Center for Advanced Studies, Moscow 123592, Russia*
[4]*Department of General Physics, Perm National Research Polytechnic University, Perm 614990, Russia*
[5]*National Research Nuclear University MEPhI, Moscow 115409, Russia*
[*]Corresponding author email address: alprokhorov33@gmail.com

S1. TEM imaging and EDX analysis

Samples visualization was implemented by TEM with a JEOL JEM-2100 microscope. For TEM studies, 20 μL of the solutions were drop-cast onto the carbon-coated copper TEM grids and dried for 30–40 min in ambient conditions.

Figure S1.1 shows transmission electron microscopy (TEM) images of nanoparticles (NPs) obtained by femtosecond laser ablation of various samples placed in water.

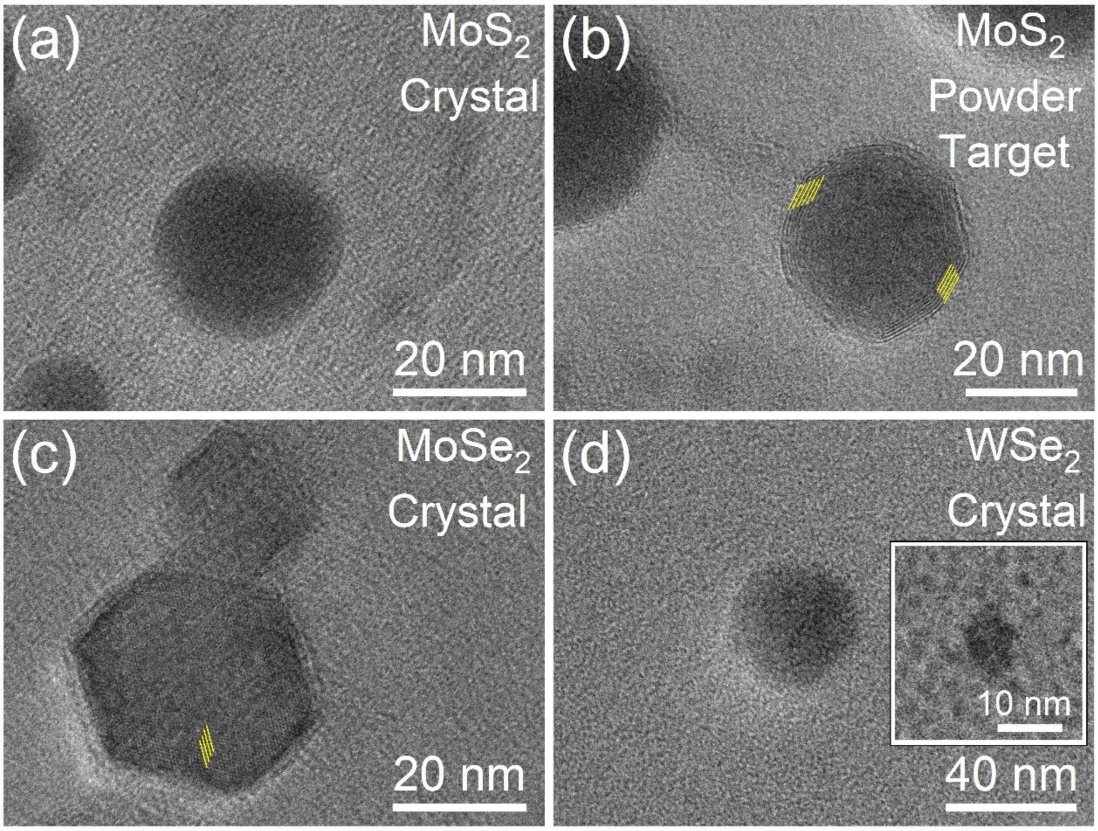

Fig. S1.1. Comparison of NP morphologies obtained by laser ablation in water from (a, c, d) crystal and (b) pressed powder targets.

Based on TEM analysis, we can assume that the NPs, obtained by ablation from crystals, have an amorphous or polycrystalline structure. In contrast, MoS_2 nanoparticles, obtained from a pressed powder target, have a core-shell structure.

After transfer of the obtained NPs from water to *N*-Methyl-2-pyrrolidone (NMP) and their subsequent ultrasonic treatment, we observe the appearance of agglomerates of spherical NPs with various sizes, see Fig. S1.2a. In samples obtained from a pressed powder target, the appearance of small (less than 10 nm in size) flat particles possessing a crystalline structure, i.e., quantum dots (QDs), is observed, see Fig. S1.2b.

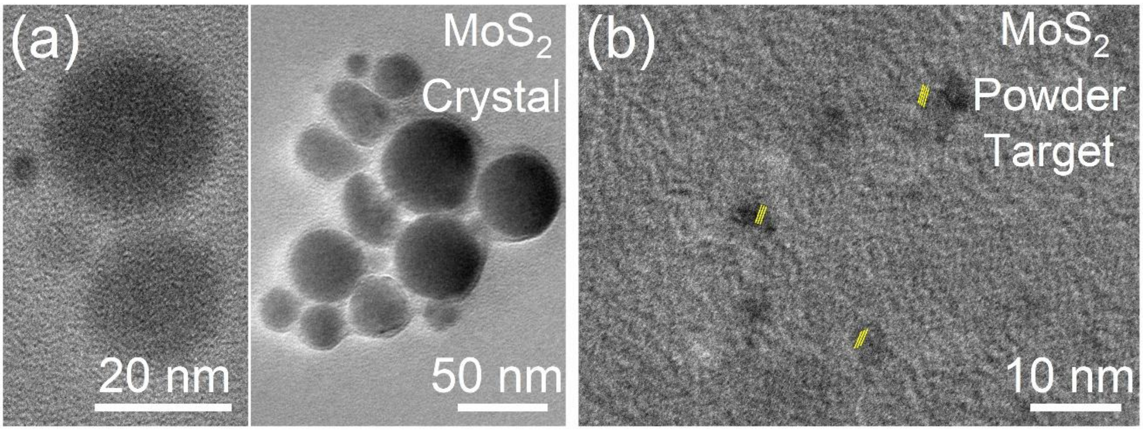

Fig. S1.2. Comparison of MoS_2 NPs and QDs obtained by two-stage method of laser ablation in water and subsequent ultrasonic treatment in NMP from the initial (a) MoS_2 crystal and (b) MoS_2 pressed powder target.

To confirm the chemical composition of NPs obtained by two-stage "fs+US" treatment of the initial samples, we used the energy dispersive x-ray (EDX) analysis, see Fig. S1.3.

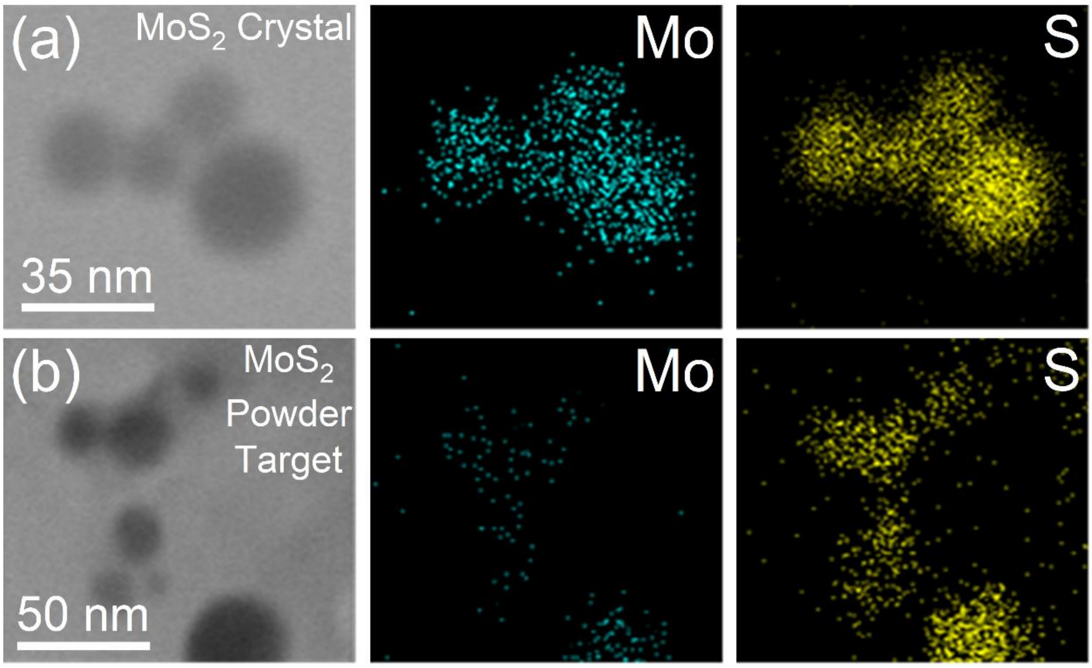

Fig. S1.3. The results of EDX analysis for NPs obtained by two-stage "fs+US" treatment of MoS_2 samples.

In general, we can conclude that the chemical composition of the obtained NPs corresponds to the initial material.

S2. Description of TMDC QDs photoluminescence and extinction properties

S2.1. Wavefunction of electron(hole)

Wavefunction of electron(hole) based on the $k \cdot p$ theory of perturbation has the following form [1]:

$$\Phi(\mathbf{r}) = \sum_n \varphi_n(\mathbf{r}) u_n(\mathbf{r}), \qquad \text{(S2.1)}$$

where summation is fulfilled over the degenerate states, $\varphi_n(\mathbf{r})$ is the slow varying envelope of wavefunction, $u_n(\mathbf{r})$ is the fast-oscillating Bloch amplitude within the unit cell.

S2.2. Time-independent Schrödinger equation

We start with the time-independent Schrödinger equation in the form [1]

$$\left[-\frac{\hbar^2 \nabla^2}{2m^*} + V(\mathbf{r})\right] \varphi(\mathbf{r}) = E\varphi(\mathbf{r}), \qquad \text{(S2.2)}$$

where $\varphi(\mathbf{r})$ is the wave function depending on the radius vector $\mathbf{r}$ of carrier position with the effective mass m^* in considered band, ∇^2 is the Laplace operator, $V(\mathbf{r})$ is the potential energy of quantum well with infinite barriers, E is the total energy of the particle, $\hbar$ is the reduced Planck's constant.

S2.3. The solutions for 3D QDs

For 3D QD, see Fig. S2.1, it is convenient to choose the following potential:

$$V(\mathbf{r}) = \begin{cases} 0, & |\mathbf{r}| \le R, \\ \infty, & |\mathbf{r}| > R. \end{cases} \qquad \text{(S2.3a)}$$

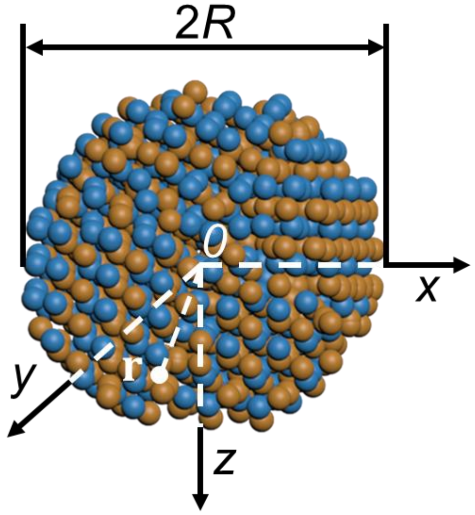

Fig. S2.1. Artistic illustration of spherical QD.

Then, we obtain the solutions of Schrödinger equation in the following forms

$$\psi_{nlm}(r) = \sqrt{\frac{2}{R^3}} \frac{j_l(k_{nl} r)}{j_{l+1}(\chi_{nl})} Y_{lm}(\Theta, \phi), \qquad \text{(S2.3b)}$$

$$E_{nl} = \frac{\hbar^2 k_{nl}^2}{2m^*},\ k_{nl}^2 = \frac{\chi_{nl}}{R}, \qquad \text{(S2.3c)}$$

where R is the sphere radius; n, l, and m are principal quantum number, orbital angular momentum quantum number, and magnetic quantum number, respectively; $j_{l(l+1)}(x)$ is the spherical Bessel function of the first kind and lth((l+1)th) order; $Y_{lm}(\Theta, \phi) = \frac{1}{\sqrt{2\pi}} e^{im\phi} \Theta_{lm}(\theta)$ is the spherical harmonic function of degree l and order m; χ_{nl} is the nth zeros of spherical Bessel function of the first kind and lth order ($j_l(\chi_{nl}) = 0$); $r = |\mathbf{r}| = \sqrt{x^2 + y^2 + z^2}$ is the absolute value of radius vector for electron (hole); θ and ϕ are the polar and azimuthal angels, respectively. The first five zeros of spherical Bessel function of the first kind and lth order ($j_l(\chi_{nl}) = 0$) are presented in

Table S2.1. Here we assume that hole energy $E_{nl}^{(h)}$ in the valence band and the electron energy $E_{n'l'}^{(e)}$ in the conduction band can be written in the following form:

$$E_{nl}^{(h)} = eE_V - \frac{\hbar^2 k_{nl}^2}{2m_e^{bl}}, \quad \text{(S2.4a)}$$

$$E_{n'l'}^{(e)} = eE_C + \frac{\hbar^2 k_{n'l'}^2}{2m_h^{bl}}, \quad \text{(S2.4b)}$$

where E_V is the energy of top of the valence band of QD (eV), E_C is the energy of the bottom of conduction band of QD (eV), $n(n')$ is the principal quantum number for hole(electron), $m_{e(h)}^{bl}$ is the effective mass of electron(hole) for isotropic (bulk) material, $l(l')$ is the orbital angular momentum quantum number for hole(electron), *e* is the absolute value of electron charge.

Table S2.1. The *n*th zero (χ_{nl}) of spherical Bessel function of the first kind and *l*th order.

	n=1	*n*=2	*n*=3	*n*=4	*n*=5
l=0	3.14	6.28	9.42	12.57	15.71
l=1	4.49	7.73	10.90	14.07	17.22
l=2	5.76	9.10	12.32	15.51	18.69
l=3	6.99	10.42	13.70	16.92	20.12
l=4	8.18	11.70	15.04	18.30	21.53

Thus, for the interband 1*S*(*e*)→1*S*(*h*) transition in QD ($n' = n = 1$, $l' = l = 0$) we obtain:

$$E_g^{3D} = eE_g^{bulk} + \frac{\hbar^2 \pi^2}{2R^2 \mu^{bl}}. \quad \text{(S2.5)}$$

where $E_g^{bulk} = E_C - E_V$ and the reduced mass of carriers μ^{bl} can be calculated as follows

$$\mu^{bl} = \frac{m_e^{bl} m_h^{bl}}{m_e^{bl} + m_h^{bl}}. \quad \text{(S2.6)}$$

The important characteristic of QD is the so-called exciton Bohr radius determined by the expression $R_b = \frac{4\pi\varepsilon_0 \hbar^2 \varepsilon_{st}}{e^2}\left(\frac{1}{m_e} + \frac{1}{m_h}\right)$, where ε_{st} is the static dielectric permittivity of QD material. In the case when QD radius *R* becomes less than R_b, the so-called strong confinement is observed in the system assuming that quantum well possess infinite barriers and neglecting the Coulomb interaction in the system. For the record, the values of exciton Bohr radius for various van der Waals (vdW) materials, as well as their monolayer thicknesses (h_{ML}), are presented in Table S2.2.

Table S2.2. The exciton Bohr radius (R_b) and monolayer thickness (h_{ML}) for four canonical TMDCs (MoS_2, WS_2, $MoSe_2$, and WSe_2).

Material	R_b, nm	h_{ML}, nm
MoS_2	2.89	0.62
WS_2	3.94	0.62
$MoSe_2$	3.96	0.645
WSe_2	4.13	0.65

S2.4. The solution for 2D TMDC QDs

For QD with cylindrical shape, see Fig. S2.2, it is convenient to choose the following potential:

$$V(\mathbf{r}) = \begin{cases} 0, & |\boldsymbol{\rho}| \le \rho_0 \text{ and } 0 \le z \le h, \\ \infty, & |\boldsymbol{\rho}| > \rho_0 \text{ and(or) } z < 0,\ z > h. \end{cases} \quad \text{(S2.7a)}$$

The solutions of equation (S2.2) for this potential have the forms:

$$\varphi_{n_z nl}(\rho) = \sqrt{\frac{2}{\pi h \rho_0^2}} \frac{J_l(k_{nl}\rho)}{J_{l+1}(\zeta_{nl})} \sin(k_{n_z} z) e^{il\theta}, \quad \text{(S2.7b)}$$

$$E_{n_z nl} = \frac{\hbar^2(k_{n_z}^2+k_{nl}^2)}{2\mu}, \quad \text{(S2.7c)}$$

where $k_{n_z} = \pi n_z/h$, $k_{nl} = \zeta_{nl}/\rho_0$; ρ_0 and h are the radius and height of cylindrical few-layers QD, respectively; $\mu = \frac{m_e m_h}{m_e+m_h}$ is the reduced effective mass. The new quantum number n_z determines the energy quantization along the *z*-axis. The parameter $J_{l(l+1)}(x)$ is the Bessel function of the first kind and *l*th((*l*+1)th) order, ζ_{nl} is the *n*th root of first kind Bessel function J_l of *l*th order ($J_l(\zeta_{nl}) = 0$); $\rho = |\boldsymbol{\rho}| = \sqrt{x^2+y^2}$ is the radial distance, i.e., absolute value of radius vector along plane parallel to the cylinder base; θ is the angle between vector $\boldsymbol{\rho}$ and *x*-axis; z is the coordinate along *z*-axis, $|\mathbf{r}| = \sqrt{\rho^2+z^2}$. The first five zeros of Bessel function of the first kind and *l*th order ($J_l(\zeta_{nl}) = 0$) are presented in Table S2.3.

Table S2.3. The *n*th zero (ζ_{nl}) of Bessel function.

	n=1	*n*=2	*n*=3	*n*=4	*n*=5
l=0	2.40	5.52	8.65	11.79	14.93
l=1	3.83	7.02	10.17	13.32	16.47
l=2	5.14	8.42	11.62	14.80	17.96
l=3	6.38	9.76	13.02	16.22	19.41

For the layered material of QD, when layers are arranged in the base plane of cylinder (see Fig. S2.2), we assume that total energy with respect to the bulk bandgap can be decoupled as follows:

$$E_{n_z nl} = E_{n_z} + E_{nl} = \frac{\hbar^2 k_{n_z}^2}{2\mu^\perp} + \frac{\hbar^2 k_{nl}^2}{2\mu^\parallel}, \quad \text{(S2.8)}$$

$$\frac{1}{\mu^{\perp(\parallel)}} = \frac{1}{m_e^{\perp(\parallel)}} + \frac{1}{m_h^{\perp(\parallel)}}, \quad \text{(S2.9)}$$

where $\mu^{\perp(\parallel)}$ is the reduced effective mass and $m_e^{\perp(\parallel)}\left(m_h^{\perp(\parallel)}\right)$ is the effective masses of electron(hole) across ($\perp$) and along ($\parallel$) layers.

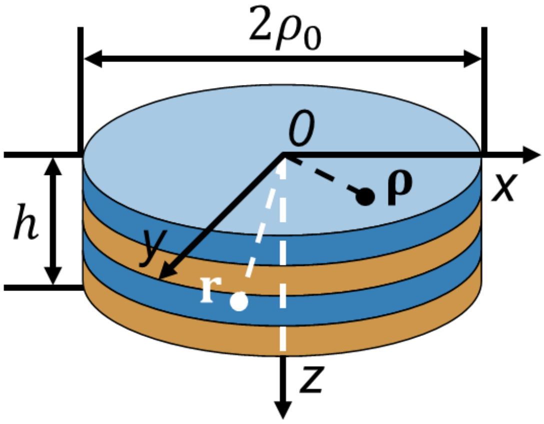

Fig. S2.2. Cylindrical QD made of layered material. Layers are oriented along base of the cylinder.

By analogue with the derivation of formula (S2.5), we suppose that hole energy $E_{nl}^{(h)}$ in the valence band and the electron energy $E_{n'l'}^{(e)}$ in the conduction band can be written in the following form:

$$E_{nl}^{(h)} = eE_V - \frac{\hbar^2 k_{n_z}^2}{2m_e^\perp} - \frac{\hbar^2 k_{nl}^2}{2m_e^\parallel}, \quad \text{(S2.10a)}$$

$$E_{n'l'}^{(e)} = eE_C + \frac{\hbar^2 k_{n'_z}^2}{2m_h^\perp} + \frac{\hbar^2 k_{n'l'}^2}{2m_h^\parallel}, \quad \text{(S2.10b)}$$

where E_V is the energy of top of the valence band of QD (eV), E_C is the energy of the bottom of conduction band of QD (eV), $n(n')$ is the principal quantum number for hole(electron), $l(l')$ is the

orbital angular momentum quantum number for hole(electron), $n_z(n'_z)$ are the principal quantum numbers along the z-axis for initial(final) state, e is the absolute value of electron charge.
Thus, the energy of bandgap transition in QD (E_g^{2D}) can be written as follows

$$E_g^{2D} = E_{n'l'}^{(e)} - E_{nl}^{(h)} = eE_g^{bulk} + \frac{\hbar^2 k_{n'_z}^2}{2m_h^{\perp}} + \frac{\hbar^2 k_{n_z}^2}{2m_e^{\perp}} + \frac{\hbar^2 k_{n'l'}^2}{2m_h^{\parallel}} + \frac{\hbar^2 k_{nl}^2}{2m_e^{\parallel}}. \quad \text{(S2.11)}$$

For $1S(e)\rightarrow 1S(h)$ transition in QD with quantum numbers $n'_z = n_z = 1$, $n' = n = 1$, $l' = l = 0$, the transition energy, which is QD bandgap energy, is equal to

$$E_g^{2D} = eE_g^{bulk} + \frac{\hbar^2\pi^2}{2\mu^{\perp}h^2} + \frac{\hbar^2 2.4^2}{2\mu^{\parallel}\rho_0^2}, \quad \text{(S2.12)}$$

where $\frac{1}{\mu^{\perp(\parallel)}} = \frac{1}{m_e^{\perp(\parallel)}} + \frac{1}{m_h^{\perp(\parallel)}}$.

S2.5. Simulation of dependences of photoluminescence wavelength on QD size and morphology

The wavelength of $1S(e)\rightarrow 1S(h)$ transition in 2D and 3D QDs can be written as follows

$$\lambda_{11}^{iD} = \frac{2\pi c\hbar}{E_g^{iD}}, \quad \text{(S2.13)}$$

where c is the speed of light in vacuum and $i = \{2,3\}$. Figure S2.3 shows the size dependences of photoluminescence wavelengths for QDs with various morphology calculated using expressions (S2.13), (S2.12), and (S2.5). We assume that 2D QDs are monolayer QDs and use data from Tables S2.2 and S2.4.

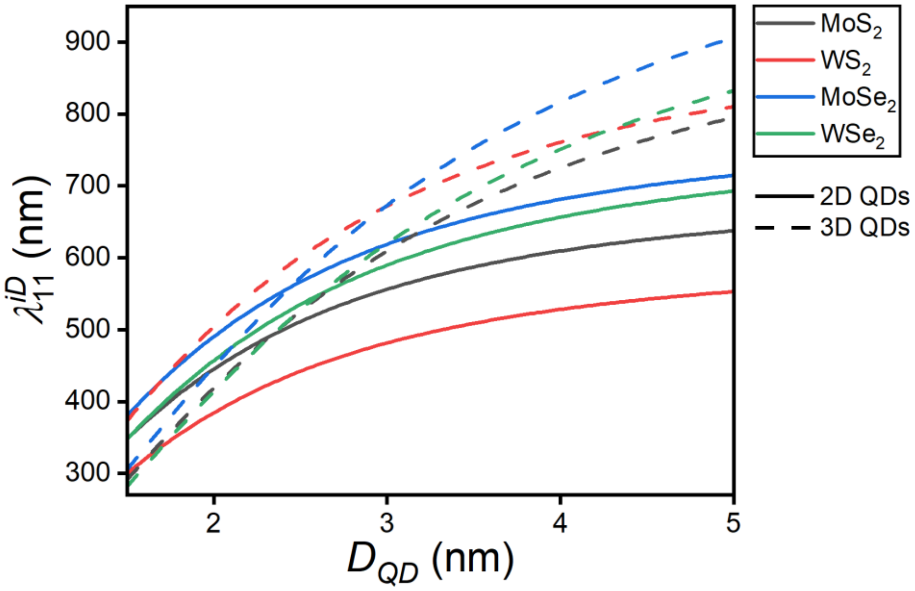

Fig. S2.3. Size dependence of monolayer 2D round-shaped QDs (solid lines) and bulk 3D QDs (dashed lines) for various vdW materials; $D_{QD} = 2R = 2\rho_0$.

S2.6. Dependence of photoluminescence wavelength on QD thickness

At this stage, we consider only thickness dependence of large area flakes of layered materials, i.e.,

$$E_g^{NML} = eE_g^{bulk} + \frac{\hbar^2\pi^2}{2\mu^{\perp}h^2}, \quad \text{(S2.14)}$$

where $h = Nh_{ML}$, N is the number of layers and h_{ML} is the thickness of monolayer, E_g^{bulk} is the indirect bulk bandgap. Although, expression (S2.14) quite good describes the experimental data for few-layer flakes of WS_2 material, except monolayer, [2], when E_g^{bulk} and $\mu^{\perp}$ are taken for the bulk, this expression does not fit the value of monolayer bandgap [2]. Probably, this is associated with the reduced mass $\mu^{\perp}$ that can significantly differ for the monolayer. Therefore, we will use

this parameter $\mu^{\perp}$ as fitting parameter in order to obtain the known (calculated or experimental) value E_g^{ML} for monolayer.

Figure S2.4 shows the dependence of parameter E_g^{NML} on the number of layers for the known vdW materials. It can be seen that E_g^{NML} value approaching to the E_g^{bulk} value upon increasing the layer number and $E_g^{NML} = E_g^{ML}$ for monolayer with N=1, which is taken from Refs. [3, 4]. Figure S2.3 shows the dependences of interband transition wavelength for monolayer 2D QDs and bulk 3D QDs made of the same materials as in Fig. S2.4. In this case, the diameter of 3D QDs and 2D QDs are equal to each other, i.e., $D_{QD} = 2R = 2\rho_0$.

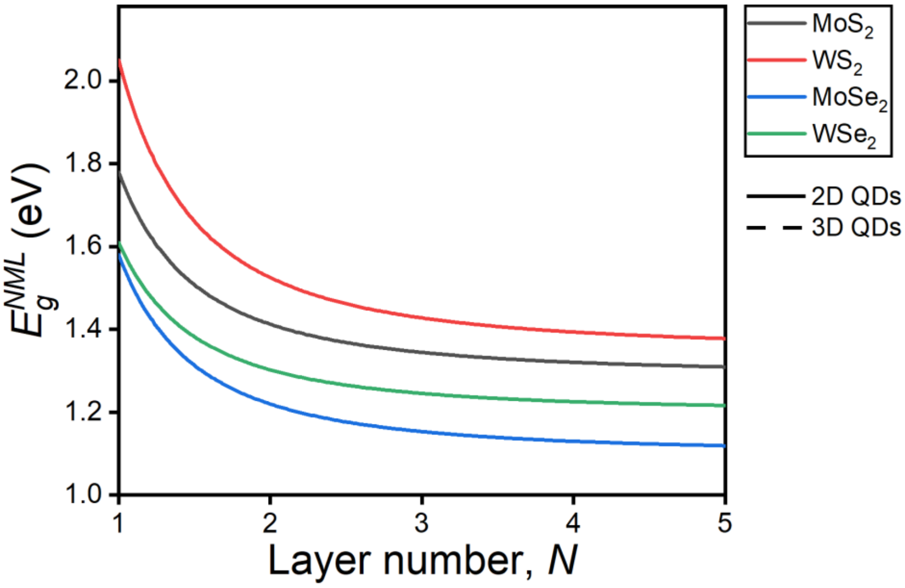

Fig. S2.4. Thickness dependence of infinite flake bandgap for various dvW materials calculated according to the formula (S2.14).

S2.7. Dipole moment of the transitions in vdW QDs

The matrix element of dipole moment for interband transition [5] can be written in the following form:

$$d_{n'n} = \int_V \varphi^*_{n_z nl} er \varphi_{n'_z n' l'}(\mathbf{r}) dV, \tag{S2.15}$$

where $r = |\mathbf{r}|$ is the distance from coordinate system origin to the electron; φ_{in} and φ_{fin} correspond to the initial $\varphi_{n_z nl}(\mathbf{r})$ ($\psi_{nlm}(\mathbf{r})$) and final $\varphi_{n'_z n' l'}(\mathbf{r})$ ($\psi_{n'l'm'}(\mathbf{r})$) states of the electron in 2D (3D) QD. Here, the integration is performed only over the volume V of QD, since we assume that there are no charge carriers outside of it.

Another energy characteristic of the interband transition is the so-called transition rate [6]:

$$W_{n'n} = \frac{2\pi A_0^2}{\hbar} |d_{n'n}|^2 \delta(E_{n'} - E_n - \hbar\omega). \tag{S2.16}$$

In the case of semiconductor QD and direct transition, we obtain:

$$W_{n'n} = \frac{2\pi A_0^2}{\hbar} |d_{n'n}|^2 \frac{1}{2\pi^2} \left(\frac{2\mu}{\hbar^2}\right)^{3/2} \left(\hbar\omega - E_g\right)^{1/2}, \tag{S2.17}$$

where E_g is the bandgap of the considered 2D or 3D QDs, ω is the angular frequency of incident light, μ equals to the reduced mass along monolayer for 2D QDs ($\mu^{\parallel}$) and bulk reduced mass for 3D QDs (μ^{bl}). The characteristic dependences of transition rate W_{11} for $1S(e) \rightarrow 1S(h)$ transition in QDs on the photon energy $E_{ph} = \hbar\omega$ and QD size for QDs with various morphology and made of various materials are presented in Fig. S2.5.

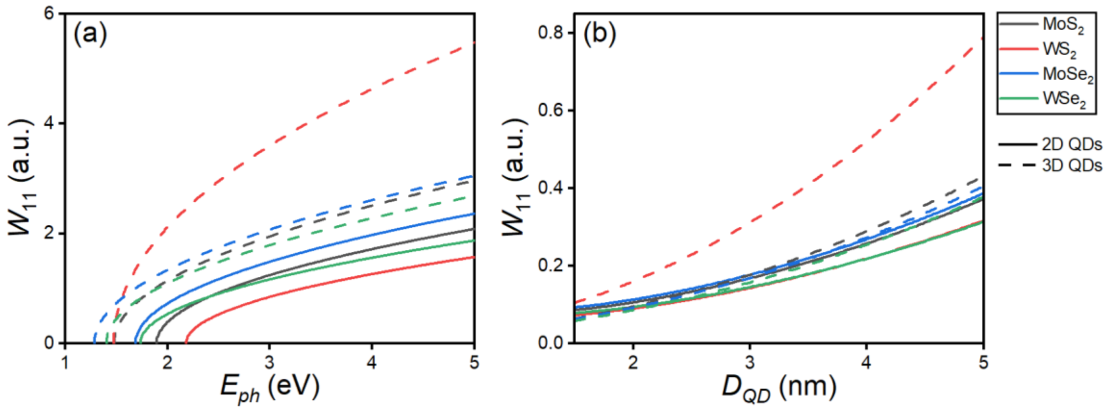

Fig. S2.5. (a) Spectral and (b) size dependences of transition rate W_{11} for monolayer 2D vdW QDs (solid curves) and 3D vdW QDs (dashed curves) with (a) fixed diameter $D_{QD} = 2R = 2\rho_0 = 6$ nm of vdW QDs and (b) different incident photon energy $\hbar\omega = 1.1E_g^{iD}$, where E_g^{iD} depends on the radius ρ_0 of 2D or R of 3D vdW QDs.

Table S2.4. Database of geometric, electronic, and optical characteristics of various TMDCs.

Parameter	MoS_2		WS_2		$MoSe_2$		WSe_2	
	bulk, 2H	monolayer	bulk, 2H	monolayer	bulk, 2H	monolayer	bulk, 2H	monolayer
Electron effective mass, m_0	0.47 [4]	0.40 [7]	0.832 [8]	0.33 [9]	0.35 [10]	0.43 [7]	0.34 [10]	0.35 [9]
Hole effective mass, m_0	0.43 [4]	0.48 [7]	0.569 [8]	0.43 [9]	0.63 [10]	0.50 [7]	0.54 [10]	0.46 [9]
Bandgap, eV	1.29 [4]	1.78 [3]	1.35 [3]	2.05 [3]	1.1 [3]	1.58 [3]	1.2 [3]	1.61 [3]
Lattice parameter (a), Å	3.19 [11]	3.13 [3]	3.18 [11]	3.14 [3]	3.32 [11]	3.29 [3]	3.32 [11]	3.31 [3]
Lattice parameter (b), Å	3.19 [11]		3.18 [11]		3.32 [11]		3.32 [11]	
Lattice parameter (c), Å	13.18 [11]		12.98 [11]		13.54 [11]		13.74 [11]	
In-plane lattice spacing (100), nm	0.27 [12]	0.27 [12]	0.27 (100) [13]	0.27 [14]	0.28 [15–17]	0.28 [15–17]	0.28 [18]	0.28 [18]
Interplanar distance (002), nm	0.62 [19]		0.62 [13]		0.645 [15]		0.65 [20, 21]	
Static permittivity		12 [22, 23]		14 [24–27]		17.4 [27]		15.6 [27]

S2.8. The spontaneous emission rate

The spontaneous radiative life time τ for the emitter in dielectric matrix can be written in the following form [28]

$$\frac{1}{\tau} = \frac{n_r \omega_{n'n}^3}{3\varepsilon_0 \pi \hbar c^3} \left|\bar{\mathbf{d}}\right|^2, \quad \text{(S2.18)}$$

where n_r is the refractive index of environment, $\omega_{n'n}$ is the transition frequency, $\bar{\mathbf{d}}$ is the k-point averaged transition dipole moment, ε_0 is the vacuum permittivity, c is the speed of light. We consider only single k point transition dipole moment, i.e., $\bar{\mathbf{d}} = d_{n'n}$.

In our case, we consider only one *k*-point (*k*=0) and $n_r = 1$, therefore, the spontaneous emission rate for the can be written as follows

$$\frac{1}{\tau} = \frac{\omega_{n'n}^3}{3\varepsilon_0 \pi \hbar c^3} \left|d_{n'n}\right|^2. \quad \text{(S2.19)}$$

Figure S2.6 show the comparison of spontaneous emission rates for 2D QDs and bulk 3D QDs synthesized from various vdW materials.

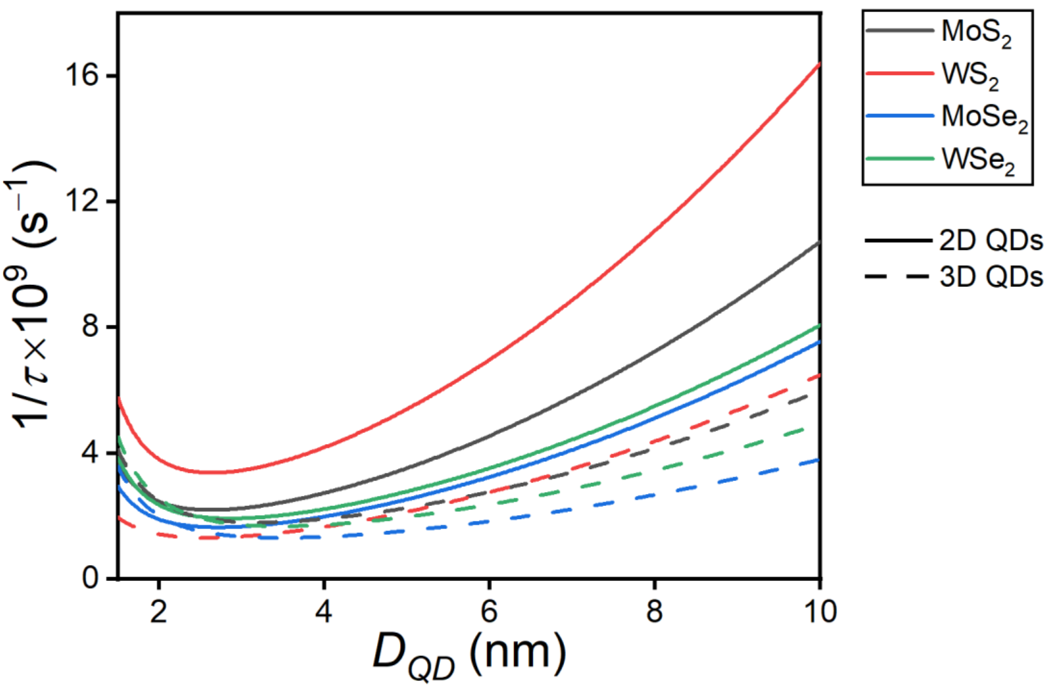

Fig. S2.6. Size dependence of spontaneous emission rate calculated using formula (S2.19) for 2D QDs (solid curves) and 3D QDs (dashed curves).

S3. Numerical processing of photoluminescence spectra

The results of fitting the experimental curves from Figs. 4 and 5 using the expressions (S2.6) and (S2.14) (taking into account that $E_g^{ML} = eE_g^{bulk} + \frac{\hbar^2\pi^2}{2\mu^{\perp}h^2}$ is the band gap of infinite monolayer) are presented in Table S3.1.

Table S3.1. Dependences of the central photoluminescence wavelengths λ_{PL} and corresponding calculated QD diameters $D_{QD} = 2R = 2\rho_0$ on the pump wavelength λ_{pump}.

λ_{pump}, nm	MoS_2 2D QD		WS_2 2D QD		$MoSe_2$ 3D QD		WSe_2 3D QD	
	λ_{PL}, nm	D_{QD}, nm	λ_{PL}, nm	D_{QD}, nm	λ_{PL}, nm	D_{QD}, nm	λ_{PL}, nm	D_{QD}, nm
**300**	356	1.54	361	1.84	373	1.73	373	1.84
**320**	395	1.72	393	2.06	387	1.78	388	1.90
**340**	401	1.75	403	2.14	398	1.82	402	1.95
**360**	471	2.17	459	2.69	457	2.03	427	2.05
**380**	488	2.30	475	2.90	481	2.12	469	2.23
**400**	498	2.38	477	2.93	495	2.18	495	2.35

Deconvolution of the PL spectra under excitation by pump wavelength 340 nm, see Figs. 4c, 4d, 5c, and 5d, was carried out based on the expression (2), i.e., Lorentz distribution function for the intensity. The central wavelength (λ_0) and full width at half maximum in the wavelength scale (FWHM_λ), as well as in the frequency scale (FWHM_ν) calculated as follows $\mathrm{FWHM}_\nu = \frac{2\pi c}{\lambda_0 - \mathrm{FWHM}_\lambda/2} - \frac{2\pi c}{\lambda_0 + \mathrm{FWHM}_\lambda/2}$, of the Lorentz distribution function corresponding to the deconvolution of PL spectra for various QDs are presented in Tables S3.2 and S3.3, where *c* is the speed of light in vacuum.

Table S3.2. The parameters λ_0, FWHM_λ, and FWHM_ν corresponding to the deconvolution peaks for PL spectra of $MoSe_2$ and WSe_2 QDs under excitation by pump wavelength 340 nm.

Peak	$MoSe_2$			WSe_2		
	λ_0, nm	FWHM_λ, nm	FWHM_ν, s^{-1}	λ_0, nm	FWHM_λ, nm	FWHM_ν, s^{-1}
1	398.0	59.3	$7.10 \cdot 10^{14}$	402.0	64.1	$7.52 \cdot 10^{14}$
2	452.6	177.4	$1.70 \cdot 10^{15}$	458.8	180.7	$1.68 \cdot 10^{15}$

Table S3.3. The parameters λ_0, FWHM_λ, and FWHM_ν corresponding to the deconvolution peaks for PL spectra of MoS_2 and WS_2 QDs under excitation by pump wavelength 340 nm.

Peak	MoS_2			WS_2		
	λ_0, nm	FWHM_λ, nm	FWHM_ν, s^{-1}	λ_0, nm	FWHM_λ, nm	FWHM_ν, s^{-1}
1	401	59.2	$6.98 \cdot 10^{14}$	403.0	73.2	$8.57 \cdot 10^{14}$
2	442.7	76.2	$7.38 \cdot 10^{14}$	456.6	85.6	$7.81 \cdot 10^{14}$
3	497.0	135.8	$1.06 \cdot 10^{15}$	511.4	134.1	$9.83 \cdot 10^{14}$

S4. Raman spectroscopy

Raman spectra were acquired with a Horiba LabRAM HR Evolution (HORIBA Ltd., Kyoto, Japan) confocal Raman microscope. For Raman studies, we drop-cast 20 μL of the colloidal solution of the NPs on the gold-coated Si substrates and dried them under ambient condition for 30–60 minutes. The excitation wavelength was 532 nm. Soptop LMPPlan 100×/0.8 infinity-corrected plan achromatic objective was used, spot size was ~1.8 μm^2. We set 600 lines/mm diffraction grating, OD1.43 optical density filter and 5×10s acquisition time. At least five spectra were acquired from each analyzed area, and their variation was slight. In particular, the spectral lines in Figs.2c, 2d, 3c, and 3d correspond to the following: MoS_2 shows E^1_{2g} and A_{1g} lines at 383

and 409 cm^{-1}, respectively [29]. WS_2 lines are located at 351 cm^{-1} (unresolved 2LA(M) and E^1_{2g}) and 420 cm^{-1} (A_{1g}) [30–32]. For $MoSe_2$, we observe E_{1g} line at 171 cm^{-1} and A_{1g} at 244 cm^{-1} [33]. WSe_2 spectra demonstrate A_{1g} mode at 180 cm^{-1} and unresolved contribution of E^1_{2g} and E_{1g} modes at 256 cm^{-1} [21, 34].

S5. IR spectroscopy of solutions of vdW QDs

To determine the presence of functional groups on the QD surface, the additional infrared spectroscopic (IR) measurements were carried out. Fourier transform infrared (FTIR) spectra were recorded on a Shimadzu IR Affinity-1S spectrometer using a Quest single-reflection attenuated total reflection (ATR) accessory (Specac), KRS-5 prism.

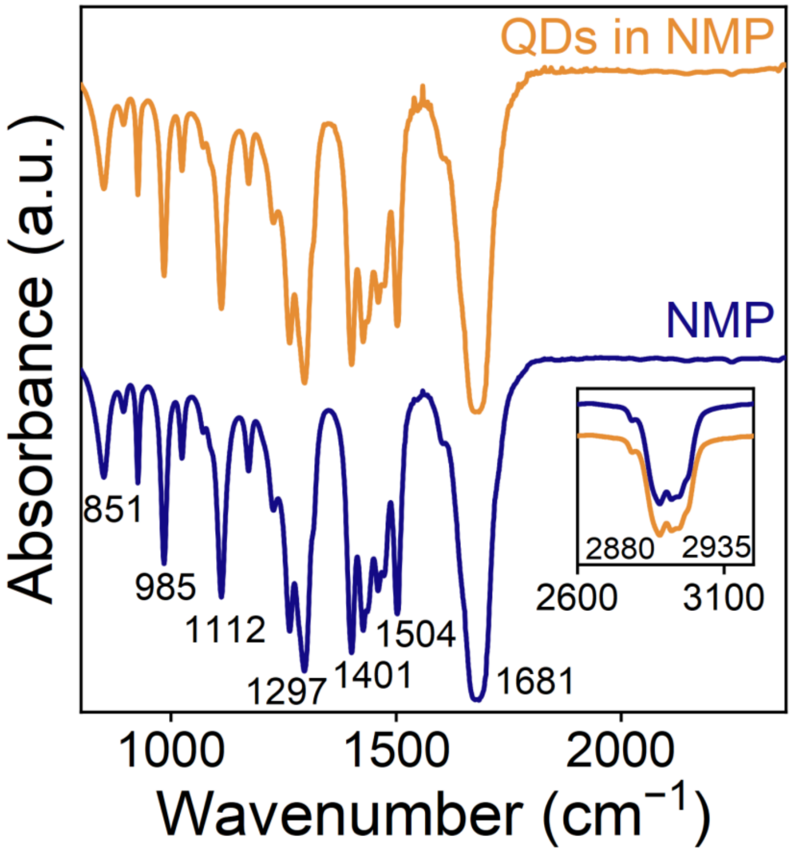

Fig. S5.1. FTIR spectra of NMP (blue curve) and MoS_2 QDs in NMP (orange curve). The inset shows the spectral range 2600-3200 cm^{-1} related to the C-H stretching vibrations. Band positions are indicated by numbers.

Figure S5.1 shows the attenuated total reflectance Fourier transform infrared (ATR-FTIR) spectrum from NMP, utilized as a medium for the fragmentation, and typical spectrum from the colloidal solution of QDs in NMP (MoS_2 QDs). Vertical offset is applied to distinguish the curves. They demonstrate well-resolved strong absorbance bands at 1681 cm^{-1} (C=O stretching), 1400-1500 cm^{-1} (C-H rocking/bending/scissoring), 1297 cm^{-1} (C-N stretching), 1112 cm^{-1} (H-C-H twisting), 985 cm^{-1} ($-CH_3$ in-plane-bending), 851 cm^{-1} (H-C-H wagging) [35]. Both spectra correspond to the well-known vibrational signature of pristine NMP [36]. Since ATR-FTIR spectroscopy is highly sensitive to the nanoparticle functionalization [37, 38], the similarity of spectra confirms the absence of ligands on QDs. This finding emphasizes a key advantage of femtosecond laser fragmentation in liquids: the ability to produce ligand-free QDs [39, 40].

S6. Absorption measurements in QD ensemble

To characterize the overall optical properties of the synthesized ensembles, optical extinction spectra were measured, see Fig. S6.1.

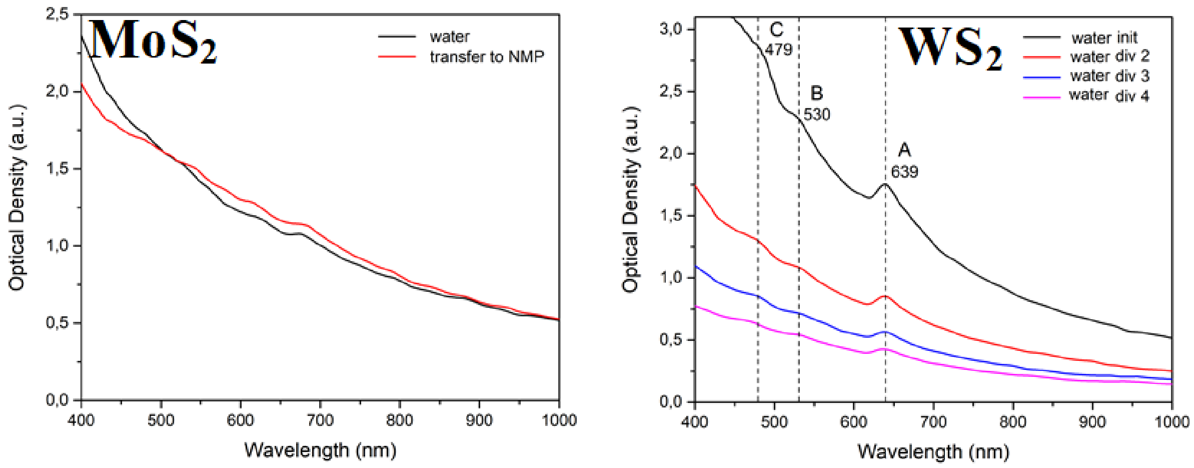

Fig. S6.1. Extinction spectra for various vdW materials after ablation in water (with varying degrees of dilution) and after transfer to NMP.

The spectra clearly show exciton features that we characterized for the WS_2 material. However, due to the wide-size dispersion and the presence of large NPs in the ensembles (Figs. 2 and 3), the extinction spectra are mainly dominated by scattering and bulk-like absorption from the larger particles, which obscures the subtle absorption features corresponding to the quantum-confined fraction (QDs). Consequently, photoluminescence (PL) spectroscopy serves as a more sensitive and selective tool for investigating the quantum confinement effects and the properties of the emissive QD fraction.

S7. Comparative analysis of synthesis efficiency, morphology, and PL intensity

While the two-stage fs+US treatment is applicable to all four TMDCs, the practicalities of synthesis and the resulting efficiencies vary considerably. The most significant distinction in the outcomes stems from the initial target morphology. The use of pressed powder targets for MoS_2 and WS_2 facilitates the formation of core-shell nanoparticles during ablation, which are subsequently exfoliated into planar 2D QDs during ultrasonication (Fig. 2). Conversely, ablation of $MoSe_2$ and WSe_2 crystals yields 3D polycrystalline or spherical NPs (Fig. 3). Furthermore, the efficiency of fragmentation varies; $MoSe_2$ demonstrates significant resistance to size reduction, resulting in the largest average particle size (29.7 nm). This morphological difference (2D vs. 3D) fundamentally determines the luminescence yield. The 2D Sulfide QDs exhibit photoluminescence across various sizes due to their few-layer nature. In contrast, the 3D Selenide QDs must be smaller than 4–5 atomic layers to overcome the bulk indirect bandgap. This is reflected in the PL intensities (Figs. 4 and 5), where WS_2 shows the highest emission (Normalized PL ~13 a.u.), significantly outperforming MoS_2 (~6.5 a.u.) and the Selenides (~5 a.u.). Thus, the synthesis pathway utilizing powder targets for 2D QD fabrication, particularly for WS_2, is identified as the most efficient route for producing bright photoluminescent ensembles using this method.

References

[1] S. V. Gaponenko, N. N. Rozanov, E. L. Ivchenko, A. V. Fedorov, A. M. Bonch-Bruevich, T. A. Vartanyan, p. G. Przhibelsky. Optics of nanostructures., Edited by A. V. Fedorov: St. Petersburg “Nedra”, 2005 - 326 pp.

[2] M. R. Molas, K. Nogajewski, A. O. Slobodeniuk, J. Binder, M. Bartos, Marek Potemski, The optical response of monolayer, few-layer and bulk tungsten disulfide, Nanoscale 9, 13128–13141 (2017). doi:10.1039/C7NR04672C

[3] A. Kumar, P. K. Ahluwalia, Electronic structure of transition metal dichalcogenides monolayers 1H-MX_2 (M = Mo, W; X = S, Se, Te) from ab-initio theory: new direct band gap semiconductors, Eur. Phys. J. B 85, 186 (2012). doi:10.1140/epjb/e2012-30070-x

[4] H. Peelaers, C. G. Van de Walle, Effects of strain on band structure and effective masses in MoS_2, Phys. Rev. B 86, 241401(R) (2012). doi:10.1103/PhysRevB.86.241401

[5] M. Asada, A. Kameyama, Y. Suematsu, Gain and intervalence band absorption in quantum-well lasers, IEEE Journal of Quantum Electronics 20, 745—753 (1984). doi:10.1109/JQE.1984.1072464

[6] M. Helm, Chapter 1 The Basic Physics of Intersubband Transitions, Semiconduct. Semimet. 62, 1-99 (1999). doi:10.1016/S0080-8784(08)60304-X

[7] S. Conti, D. Neilson, F. M. Peeters, A. Perali, Transition Metal Dichalcogenides as Strategy for High Temperature Electron-Hole Superfluidity, Condens. Matter 5, 22 (2020). doi:10.3390/condmat5010022

[8] D. Wickramaratne, F. Zahid, R. K. Lake, Electronic and thermoelectric properties of few-layer transition metal dichalcogenides, J. Chem. Phys. 140, 124710 (2014). doi:10.1063/1.4869142

[9] J. Chang, L. F. Register, S. K. Banerjee, Ballistic performance comparison of monolayer transition metal dichalcogenide MX2 (M = Mo, W; X = S, Se, Te) metal-oxide-semiconductor field effect transistors, J. Appl. Phys. 115, 084506 (2014). doi:10.1063/1.4866872

[10] D. Nayak, R. Thangavel, Comparative study of the electronic and photocatalytic properties of bulk and monolayer MX2: A TB-mBJ study, Mater. Sci. Eng. B 264, 114944 (2021). doi:10.1016/j.mseb.2020.114944

[11] https://next-gen.materialsproject.org/

[12] R. Wei, H. Zhang, X. He, Z. Hu, X. Tian, Q. Xiao, Z. Chen, J. Qiu, Versatile preparation of ultrathin MoS_2 nanosheets with reverse saturable absorption response, Opt. Mater. Express 5, 1807–1814 (2015). doi:10.1364/OME.5.001807

[13] C. S. Rout, P. D. Joshi, R. V. Kashid, D. S. Joag, M. A. More, A. J. Simbeck, M. Washington, S. K. Nayak, D. J. Late, Superior Field Emission Properties of Layered WS_2-RGO Nanocomposites, Sci. Rep. 3, 3282 (2013). doi:10.1038/srep03282

[14] C. Lan, R. Zhang, H. Wu, S. Wen, R. Zou, X. Kang, C. Li, J. C. Ho, Y. Yin, Y. Liu, Enhanced epitaxial growth of two-dimensional monolayer WS_2 film with large single domains, Appl. Mater. Today 25, 101234 (2021). doi:10.1016/j.apmt.2021.101234

[15] V. Babacic, D. Saleta Reig, S. Varghese, T. Vasileiadis, E. Coy, K.-J. Tielrooij, B. Graczykowski, Thickness-Dependent Elastic Softening of Few-Layer Free-Standing $MoSe_2$, Adv. Mater. 33, 2008614 (2021). doi:10.1002/adma.202008614

[16] Z. Chen, H. Liu, X. Chen, G. Chu, S. Chu, H. Zhang, Wafer-Size and Single-Crystal $MoSe_2$ Atomically Thin Films Grown on GaN Substrate for Light Emission and Harvesting, ACS Appl. Mater. Interfaces 8, 20267–20273 (2016). doi:10.1021/acsami.6b04768

[17] P. B. James, M. T. Lavik, The crystal structure of $MoSe_2$, Acta Crystallogr 16, 1183 (1963). doi:10.1107/S0365110X6300311X

[18] R. Guo, Y. Han, C. Su, X. Chen, M. Zeng, N. Hu, Y. Su, Z. Zhou, H. Wei, Z. Yan, Ultrasensitive room temperature NO_2 sensors based on liquid phase exfoliated WSe_2 nanosheets, Sens. Actuators B Chem. 300, 127013 (2019). doi:10.1016/j.snb.2019.127013 https://doi.org/10.1016/j.snb.2019.127013

[19] X. Zhang, H. Li, H. Yang, F. Xie, Z. Yuan, L. Zajickova, W. Li, Phase-Engineering of 1T/2H Molybdenum Disulfide by Using Ionic Liquid for Enhanced Electrocatalytic Hydrogen Evolution, ChemElectroChem 7, 3347–3352 (2020). doi:10.1002/celc.202000745
[20] A. Sierra-Castillo, E. Haye, S. Acosta, C. Bittencourt, J.-F. Colomer, Synthesis and Characterization of Highly Crystalline Vertically Aligned WSe_2 Nanosheets, Appl. Sci. 10, 874 (2020). doi:10.3390/app10030874
[21] R. Konar, R. Perelshtein, E. Teblum, M. Telkhozhayeva, M. Tkachev, J. J. Richter, E. Cattaruzza, A. P. Charmet, P. Stoppa, M. Noked, G. D. Nessim, Scalable Synthesis of Few-Layered 2D Tungsten Diselenide (2H-WSe_2) Nanosheets Directly Grown on Tungsten (W) Foil Using Ambient-Pressure Chemical Vapor Deposition for Reversible Li-Ion Storage, ACS Omega 5, 19409–19421 (2020). doi:10.1021/acsomega.0c01155
[22] G. A. Ermolaev, Y. V. Stebunov, A. A. Vyshnevyy, D. E. Tatarkin, D. I. Yakubovsky, S. M. Novikov, D. G. Baranov, T. Shegai, A. Y. Nikitin, A. V. Arsenin, V. S. Volkov, Broadband optical properties of monolayer and bulk MoS_2, NPJ 2D Mater. Appl., 4, 21 (2020). doi:10.1038/s41699-020-0155-x
[23] B. Mukherjee, F. Tseng, D. Gunlycke, K. K. Amara, G. Eda, E. Simsek, Complex electrical permittivity of the monolayer molybdenum disulfide (MoS_2) in near UV and visible, Opt. Mater. Express 5, 447–455 (2015). doi:10.1364/OME.5.000447
[24] S. Sharma, J. Singh, S. Bhagat, M. Singh, S. Sharma, Size-tunable photoluminescence from WS_2 nanostructures, Mater. Res. Express 5, 045047 (2018). doi:10.1088/2053-1591/aabdcd https://doi.org/10.1088/2053-1591/aabdcd
[25] C. Hsu, R. Frisenda, R. Schmidt, A. Arora, S. Michaelis de Vasconcellos, R. Bratschitsch, H. S. J. van der Zant, A. Castellanos-Gomez, Thickness-Dependent Refractive Index of 1L, 2L, and 3L MoS_2, $MoSe_2$, WS_2, and WSe_2, Adv. Optical Mater. 7, 1900239 (2019). doi:10.1002/adom.201900239
[26] G.-H. Jung, S.J. Yoo, Q-H. Park, Measuring the optical permittivity of two-dimensional materials without *a priori* knowledge of electronic transitions, Nanophotonics 8, 263–270 (2019). doi:10.1515/nanoph-2018-0120
[27] A. Laturia, M. L. Van de Put, W. G. Vandenberghe, Dielectric properties of hexagonal boron nitride and transition metal dichalcogenides: from monolayer to bulk, npj 2D Mater. Appl. 2, 6 (2018). doi:10.1038/s41699-018-0050-x
[28] D. Dhaliah, Y. Xiong, S. M. Griffin, G. Hautier, First principles study of the T-center in Silicon, Phys. Rev. Materials 6, L053201 (2022). doi:10.1103/PhysRevMaterials.6.L053201
[29] I. A. Zavidovskiy, I. V. Martynov, D. I. Tselikov, A. V. Syuy, A. A. Popov, S. M. Novikov, A. V. Kabashin, A. V. Arsenin, G. I. Tselikov, V. S. Volkov, A. D. Bolshakov, Leveraging Femtosecond Laser Ablation for Tunable Near-Infrared Optical Properties in MoS_2-Gold Nanocomposites, Nanomaterials 14, 1961 (2024). doi:10.3390/nano14231961
[30] E. P. dos Santos, F. L. R. Silva, R. N. Gontijo, J. M. Alves, M.-R. Ammar, C. Fantini, Temperature dependence of the double-resonance Raman bands in bilayer WSe_2, Vib. Spectrosc. 110, 103117 (2020). doi:10.1016/j.vibspec.2020.103117
[31] Abid, P. Sehrawat, S. S. Islam, Broadband photodetection in wide temperature range: Layer-by-layer exfoliation monitoring of WS_2 bulk using microscopy and spectroscopy, J. Appl. Phys. 125, 154303 (2019). doi:10.1063/1.5080922
[32] A. P. S. Gaur, S. Sahoo, J. F. Scott, R. S. Katiyar, Electron–Phonon Interaction and Double-Resonance Raman Studies in Monolayer WS_2, J. Phys. Chem. C 119, 5146–5151 (2015). doi:10.1021/jp512540u
[33] D. Nam, J.-U. Lee, H. Cheong, Excitation energy dependent Raman spectrum of $MoSe_2$, Sci. Rep. 5, 17113 (2015). doi:10.1038/srep17113
[34] N. D. Boscher, C. J. Carmalt, I. P. Parkin, Atmospheric pressure chemical vapor deposition of WSe_2 thin films on glass—highly hydrophobic sticky surfaces, J. Mater. Chem. 16, 122–127 (2006). doi:10.1039/B514440J

[35] W. Xu, H. Wang, Y. Tao, X. Zheng, The structural organization of *N*-methyl-2-pyrrolidinone in binary mixtures probed by Raman spectroscopy: Experimental and quantum chemical results, J. Raman Spectrosc. 49, 362–371 (2018). doi:10.1002/jrs.5291
[36] H. C. Yau, M. K. Bayazit, J. H. G. Steinkea, M. S. P. Shaffer, Sonochemical degradation of N-methylpyrrolidone and its influence on single walled carbon nanotube dispersion, Chem. Commun. 51, 16621–16624 (2015). doi:10.1039/C5CC06526G
[37] I. A. Mudunkotuwa, A. A. Minshid, V. H. Grassian, ATR-FTIR spectroscopy as a tool to probe surface adsorption on nanoparticles at the liquid–solid interface in environmentally and biologically relevant media, Analyst 139, 870–881 (2014). doi:10.1039/c3an01684f
[38] B. Panella, A. Vargas, D. Ferri, A. Baiker, Chemical Availability and Reactivity of Functional Groups grafted to Magnetic Nanoparticles monitored In situ by ATR-IR Spectroscopy, Chem. Mater. 21, 4316–4322 (2009). doi:10.1021/cm901665f
[39] L. S. De Bortoli, C. R. Vanoni, C. L. Jost, D. Z. Mezalira, M. C. Fredel, Stable and ligand-free gold nanoparticles produced by laser ablation as efficient electrocatalysts for electrochemical sensing of dopamine, J. Electroanal. Chem. 947, 117744 (2023). doi:10.1016/j.jelechem.2023.117744
[40] D. Zhang, B. Gökce, S. Barcikowski, Laser Synthesis and Processing of Colloids: Fundamentals and Applications, Chem. Rev. 117, 3990–4103 (2017). doi:10.1021/acs.chemrev.6b00468
